# The experience of the self in Canadian youth living with anxiety: A qualitative study

**DOI:** 10.1371/journal.pone.0228193

**Published:** 2020-01-31

**Authors:** Roberta L. Woodgate, Ketan Tailor, Pauline Tennent, Pamela Wener, Gary Altman

**Affiliations:** 1 College of Nursing, Rady Faculty of Health Sciences, University of Manitoba, Winnipeg, Manitoba, Canada; 2 Counseling and Psychological Services, University of Kansas, Lawrence, Kansas, United States of America; 3 Department of Occupational Therapy, Rady Faculty of Health Sciences, College of Rehabilitation Sciences, University of Manitoba, Winnipeg, Manitoba, Canada; 4 Department of Psychiatry, Rady Faculty of Health Sciences, Max Rady College of Medicine, University of Manitoba and PsycHealth Centre, Winnipeg, Manitoba, Canada; University of Sao Paulo Medical School, BRAZIL

## Abstract

**Background:**

Anxiety can create serious disruption in the life and mind of youth who are affected. Youth living with anxiety suffer a wealth of physical and psychological challenges, yet little is known about how anxiety influences the sense of the self. The purpose of this research was to explore the experience of the self in a sample of Canadian youth living with anxiety.

**Materials and methods:**

The qualitative research approach of hermeneutic phenomenology was used. The sample consisted of 58 Canadian youth with anxiety, 44 females and 14 males between the ages of 10 and 22. Youth took part in open-ended interviewing, ecomaps, and photovoice. Data analysis followed a staged process, informed by Max van Manen. All sources of data were included in the analysis to form thematic statements.

**Results:**

Entering into the lifeworld of youth revealed that they suffered deeply. A fractured sense of self underlined their experience, setting up for a great deal of self-scrutiny and a lack of self-compassion. They experienced a profound sense of responsibility for others at the loss of being-there-for-oneself. Navigating their social sphere presented an additional challenge. However, youth were genuinely interested in self-discovery through awareness and reflection.

**Conclusions:**

The phenomenological accounts by youth on living with anxiety reinforce the challenges they experienced within themselves that give rise to a great deal of inner turmoil. Care and support to youth with anxiety requires an understanding of the ways in which the self may be fractured by their experiences with anxiety. Providing young people with an opportunity to share with others who had similar lived experiences can serve to contribute to a sense of healing for youth, while also providing a safe space in which young people can let down their guard and openly acknowledge or share their experiences without fear of stigmatization.

## Introduction

The Public Health Agency of Canada has found that as many as 25% of youth (15 to 24 years) meet the criteria for a psychiatric disorder in Canada [1]. The most commonly diagnosed condition in Canada are anxiety disorders, which affects approximately 10% of children and adults across the country [2]. A meta-analysis found that in the United States, the prevalence of an anxiety disorder was 11% among youth aged 13 through 18 [3]. The experience of anxiety is by no means unidimensional, as reflected in the numerous diagnostic classifications set forth by the American Psychiatric Association for anxiety-related conditions alone (seven diagnoses total) [4]. Adding to the range of how anxiety is experienced is that comorbidity in diagnoses is common. Comorbidity among the anxiety disorders themselves is frequent [5] as is the co-occurrence of depression [6]. Anxiety-affected youth also tend to be at greater risk for suicidal ideation and behaviour than their peers without an anxiety diagnosis [7]. Although youth with anxiety suffer a wealth of physical and psychological challenges, little is known about how anxiety affects the sense of the self.

## Literature review

### Anxiety in youth

Anxiety can be disruptive to the life and the mind of the sufferer. With an early onset (median age of onset is 6 years old), anxiety disorders are chronic, disabling conditions that can negatively impact youth’s life at home and school, and can put youth at risk for adult psychiatric disorders [8–16]. Over 95% of youth living with anxiety experience at least one somatic symptom including headache, stomachache, head cold or sniffles, sleeplessness, or feelings of drowsiness or sleepiness [17]. The hallmark of anxiety in young people appears to be worry [18]. Anxiety-affected youth may worry about seemingly everyday tasks as well as the sights and sounds of everyday life [19], about feeling different, and about their social relationships. They may also worry about their health and the health of others, their performance in school or extracurricular activities, as well as fears as it relates to disasters (e.g., hurricanes) and other external events that could threaten their well-being [20].

Youth living with anxiety, in comparison to their peers, demonstrate a heightened level of negative emotionality in situations that present little or no “real” threat [21], although their reactions to those situations are very real. The ways in which they cope with these often-exaggerated feelings have important implications for how their anxiety might present. Earlier work by Suveg and Zeman [22] found that children with an anxiety disorder who experienced worry and sadness with heightened intensity, also lacked confidence in their ability to regulate those states. A hermeneutic-phenomenological study conducted by Hjeltnes et al. [23] supports this position, in that the young adults in their sample described their anxiety as “an uncontrollable inner force” that led them to fear their own feelings. They believed that they did not have the “tools” to handle their anxiety, and they dreaded the relational encounters that could provoke their anxiety. Work by Suveg et al. [24] revealed that less emotional expression and a greater tendency to express negative feelings in the family makes it more difficult for youth with an anxiety disorder to understand their emotions and learn to regulate them.

### The self

Phenomenological and psychoanalytic traditions consider the self to be the center point of experiencing, and with any rupture to the self, there is the potential to elicit a great deal of suffering. Concepts such as self-esteem, self-efficacy, and self-image have been studied in youth living with anxiety. Findings from this work tend to reveal a negative association between each of these concepts and the experience of anxiety; in other words, anxiety appears to rise where there is a disruption in the self [25–27]. Quite notably, O’Connor et al. [28] conducted a systematic review on how a psychiatric diagnosis (inclusive but not exclusive to disorders of anxiety) can influence the self-concept (one’s beliefs about oneself) and social identity (self-concept that derives from membership in social groups) of children and adolescents. However, while a diagnosis has the potential to devalue children’s self-concept and contribute to social alienation, invalidation and stigmatization, O’Connor et al. [28] noted that it can lead to understanding, validation, legitimation, and enhancement of the self as well as facilitate identification and acceptance within their social sphere.

Hjeltnes et al. [23] have revealed that the anxiety-affected young adults of their sample experienced themselves as “fundamentally flawed, weak, and unworthy…they struggled with shame, inferiority, and self-criticism, finding it hard to be assertive or self-compassionate” (p. 8). The beliefs these young people held of themselves made it difficult to be with others due to a relentless self-criticism, and while the youth were aware of their lack of compassion for themselves, it was challenging to generate self-compassion when anxiety emerged. Leavey [29] further set out to describe in their sample of youth the process of “becoming, living with, and recovering from a mental illness,” which included but was not limited to an anxiety disorder. Youth noted how they had internalized the stereotypical views that society held around mental illness (e.g., mental illness as self-inflicted), which was depreciating for them. Participants described experiencing themselves as foreign, and in some cases undergoing a complete loss of the self. Youth nonetheless described a time in which they were able to understand that their mental illness was not their fault; they could distinguish the self from the illness, allowing them to accept themselves more readily [29].

To our knowledge, only three studies have attempted to understand the attention given to self among youth living with anxiety. Takishima-Lacasa et al. [30] considered the notion of self-consciousness in youth living with anxiety. Using the Self-Consciousness Scales among a school sample, they revealed that public self-consciousness (e.g., appearance, manners) and private self-consciousness (e.g., one’s own feelings and beliefs) were significantly related to social anxiety in their participants. Drawing on lived accounts from online questionnaires and interviews, Boyle [31] found that social anxiety in youth was apparent in the ways that “micro-textures” (e.g., unthinking practices such as crossing the road) aroused in youth a “…crippling self-consciousness and palpable awareness of the occupation of social space” (p. 5). Last, Mor et al. [32] set out to examine the association between negative affect and self-focus in adolescents. Using diary measures completed by adolescents over the course of a three-day period, they found that adolescents with an anxiety disorder reported a greater amount of momentary negative affect, although this was independent of their focus on self.

While the work to date has yielded understandings on the nature of anxiety-affected youth’s attention to self, the work is still in its infancy. Accordingly, the purpose of this study was to explore the experience of the self in a sample of Canadian youth who suffered from anxiety. The study builds on the nascent literature by engaging a hermeneutic phenomenology approach, which varies from the majority of available studies in that it seeks to understand and describe of the self as it is lived, and not merely in terms of a heuristic concept.

## Methods

### Study design

The qualitative research approach of hermeneutic phenomenology was used as it afforded the opportunity to understand how living with anxiety shaped youth’s sense of self from their frames of reference and experiences of reality. In addition to open-ended interviews, the arts-based participatory data collection methods of ecomaps and photovoice were used to gain an understanding of the lived experiences of participating youth.

### Participants

Youth with a primary diagnosis of one or more anxiety disorders, including separation anxiety, social anxiety disorder, generalized anxiety disorder, and specific phobia were invited to take part in the study. Their parents were also invited to participate in the study. Youth were recruited via invitation letters from hospital-based programs that deal specifically in the treatment of anxiety as well as from youth centres, teen clinics, schools, and social media via an invitation poster. Parents completed the Anxiety Disorders Interview Schedule for Parents-IV, the most widely used diagnostic interview schedule for assessing the presence of anxiety disorders [33, 34]. In order to capture the complexity, depth, and variation of youth living with an anxiety disorder, participants were selected using the the maximum variation technique of purposive sampling [35]. Recruitment was stopped once redundancy or data saturation was achieved.

The present study included 58 Canadian youth who carried a primary diagnosis of one or more anxiety disorders. Ages of the youth ranged from 10 to 22 years at the time of the study with a mean age of 14.5 years. This broad range of age was an attempt to capture developmental differences and a diverse range of experiences among young people diagnosed with anxiety disorders. 44 participants identified as female and 14 as male.

### Ethical considerations

Ethical approval was obtained from the University of Manitoba Education/Nursing Research Ethics Board (ethics board approval number E2011:094). For participants over 18 years of age, written informed consent was obtained and for those under 18 years, assent and written informed consent from youth and parents respectively, was obtained.

### Data collection

Open-ended, face-to-face interviews were conducted that afforded the opportunity to consider the most salient aspects of the youth’s experience of living with anxiety. Youth were engaged as “informants” who are knowledgeable persons about the phenomenon under study, in this case that of anxiety [36]. Repeated interviews are an essential feature of a hermeneutic phenomenology in that they allow for follow-up questions that add to topics and themes discovered in the initial interview. More than one interview also affords participants adequate time to reflect and share their stories. Accordingly, two interview sessions were carried out. Interviews were conducted face-to-face at a private location that was most convenient and comfortable to participants (i.e., mainly in their homes) and involved the youth participant and a research assistant trained by the first author. Interviews varied from one half-hour to three hours in length. Field notes were recorded after each interview, detailing the context of the interview. To preserve their authenticity, all interviews were digitally recorded and transcribed verbatim.

In the first interview session, the opening question was, “Can you please tell me a little bit about yourself?” followed by questions meant to get at what it is like to be a youth living with anxiety and how it influences their sense of self. To facilitate the interviewing process, an interview guide with open-ended questions was used (S1 Interview). Great care was taken that the questions remained close to youth’s experience. Ecomaps were used to supplement material in the first interview while photovoice was used to supplement the second. Ecomaps are graphic portrayals of objects (people, activities, places) that play a part in the youth’s lives (represented by circles); they attempt to illustrate the degree of connection between themselves and these objects represented by different types of lines (e.g., a squiggly line indicating a weak, stressful connection) [37]. Before the initial interview, youth were asked to draw an ecomap that was then used as a point of discussion during the interview. Specifically, they were asked to draw a circle representing themselves and circles that represent people, activities, and places that are and have been a part of their lives in the context of living with anxiety. They were then ask to draw different types of lines that represent the nature of the connection between themselves and the other circles. Throughout the interview, the youth’s ecomap was used to facilitate discussion along with interview strategies including the use of silence, calls for examples, and simple questions to expand on youth’s narratives.

The participatory method of photovoice was applied prior to the second interview, which allowed for experiential descriptions in phenomenology [38]. Viewed as an unobtrusive research strategy of entering the worlds of individuals, photovoice involves individuals taking photographic images to document and think about issues important to them [39–42]. Each youth was given a digital camera at the completion of the first interview and asked to take pictures over a period of three to four weeks of people (if they obtained permission from them), objects, places or events that portrayed their experience of living with anxiety. During the second open-ended interview (S2 Interview), youth were asked questions based on the SHOWeD method [43], which encourages discussion with participants on the meaning of the photos. In this case, youth were asked to describe what the photos meant to them in terms of living with anxiety and thereby, contributing to youth’s narratives [39, 41].

### Data analysis

A staged approach, via a hermeneutic phenomenology informed by Max van Manen [38] was applied to analyze the data. Analysis started the moment youth participants began describing their experiences, which necessitated the research team to become fully immersed in the data. Each interview was listened to after the interview was conducted, and then once transcribed, was read and reread. Units of meanings of the youth’s experiences emerged by selecting appropriate phrases and capturing specific statements. Attention was given to identifying data as it related specifically to youth’s experience of the self. Entering into the lifeworld of youth revealed striking experiences of the self in youth living with anxiety. Notions of self were considered in the tradition of van Manen [44] who draws on the phenomenology of the body [45–48]. van Manen [44] starts from the position that it is the “broken, disrupted, or disturbed relation with the body that seems characteristic of almost all experience of injury or illness” (p. 3). It should be noted that the body here is not understood purely as the material, but rather in terms of the material and the spiritual and hence encompassing one’s sense of *self* [46].

As units of meanings were identified and defined, they and supporting data were then entered and organized into a table of contents created in Microsoft Word. The units of meanings were then grouped together to form thematic statements by the first three authors. The units of meanings and thematic statements were further reviewed until essential themes representing the youth’s experiences were finalized. All disagreements related to the essential themes were resolved by discussion involving all five authors (i.e., research team) until consensus was reached. Ongoing discussions among the research team throughout the study also afforded the researchers the opportunity to take a reflexive stance on their own worldview, address their assumptions, and deal with any biases. Contributing to a more inclusive understanding of the experiences of youth were the ecomaps and photographs, which served as graphic or visual representations of the text-based findings [39, 41]. During the second interview sessions, preliminary themes were shared and discussed with youth participants, which added to and confirmed the researchers’ interpretations. As well, a youth advisory committee involving six youth from the study was formed to provide advice on the finalized themes. Finally, measures including prolonged engagement with participants and data, careful line-by-line analysis of the transcripts, and detailed memo writing were in place to enhance the trustworthiness of the research process [49, 50].

### Findings

Six core themes emerged relative to the youth’s experience of the self, including *the self as fractured*, *being-there-for-oneself*, *discovery of the self*, *masking the self*, *trust in the self*, and *transcendental self*.

### The self as fractured

Youth’s descriptions of self gave the impression of something being fundamentally wrong with the self–a “fractured self.” This was reflected in their descriptions of being “broken,” “a ghost,” and “a nothing,” descriptions that were influenced by the experience of anxiety that possessed the character of an encumbrance. It is interesting to note that the experience of fragmentation within the self is not unique to youth with anxiety and is common to young people in the West who have endured a psychological trauma such as incest [51], as well as a physical illness such as cancer [49]. The experience appeared to be unanimously agonizing for youth and heard in their accounts that were parallel to van Manen’s [44] explorations, gave rise to an existential questioning about one’s physical form, wherein a detached curiosity about the body might emerge:

***Interviewer***: *What makes you think you’re a better ghost than a human being [as noted in the youth’s photograph of quote]*?

***Youth***: *Because after a really long time of feeling like such a failure and like feeling invisible and feeling like you’re nothing*, *it’s kind of like you are a ghost*. *So*

*I feel there’s a lot of times where I feel like what if I did actually die when I tried to kill myself*, *what if I actually am a ghost*, *am I actually here or is this just*, *I don’t know I always feel like that*.

Female, 17 years

The experience of a fractured self became apparent through the ways in which youth related to themselves, which was in some cases characterized by a relentless internal battle. This battle created a great deal of suffering for youth; they scrutinized themselves to a point of utter confusion, and in some cases into a state of quiescence. The quotes below illustrate some of these points:

*It’s [experience of anxiety] like a feeling*, *it is kind of like a mix between anger and like apathetic*, *like apathy to myself and like stuff like that*. *It is just kind of like a mix of a bunch of things that like making my mood kind of like*, *it’s like when you put a cat inside a bag and they start like clawing at it*.

Male, 14 years

*But like I’ll go back and forth and fight with myself trying to like love them and hate them at the same time*. *It is like non-stop*, *like I fight with myself too much*. *Just anything*! *Like if I were to go to a store*, *I’ll try and get up*, *apparently I’ll sit back down and be like I don’t want to and then I want to again*. *Like I have my money ready*, *I’m all dressed and be like I’m ready to go and it’s like as soon as I get my shoes on*, *I’ll sit back down*. *I’ll be like screw it*.

Female, 14 years

*It feels kind of like all the happiness is gone out of you*, *it feels like a war against yourself where to just fight against to try to get some sort of happiness in there*, *but all that comes out is sweat and tears and anger and aggressiveness*. *And the aggressiveness usually you bring it out on somebody else but*, *but you’re not*, *you’re not mad at anybody else*, *you’re mad at yourself*. *I’ve figured out that you’re doing a war against yourself*, *your trying to fight for everything you know against yourself and it*, *and just this random piece of anger comes and tries…it’s like a fever it tries to come and infect your whole body…Um and you’re trying to fight against it and it makes you so aggressive and you just hate yourself*.

Female, 10 years

**Fig 1:**
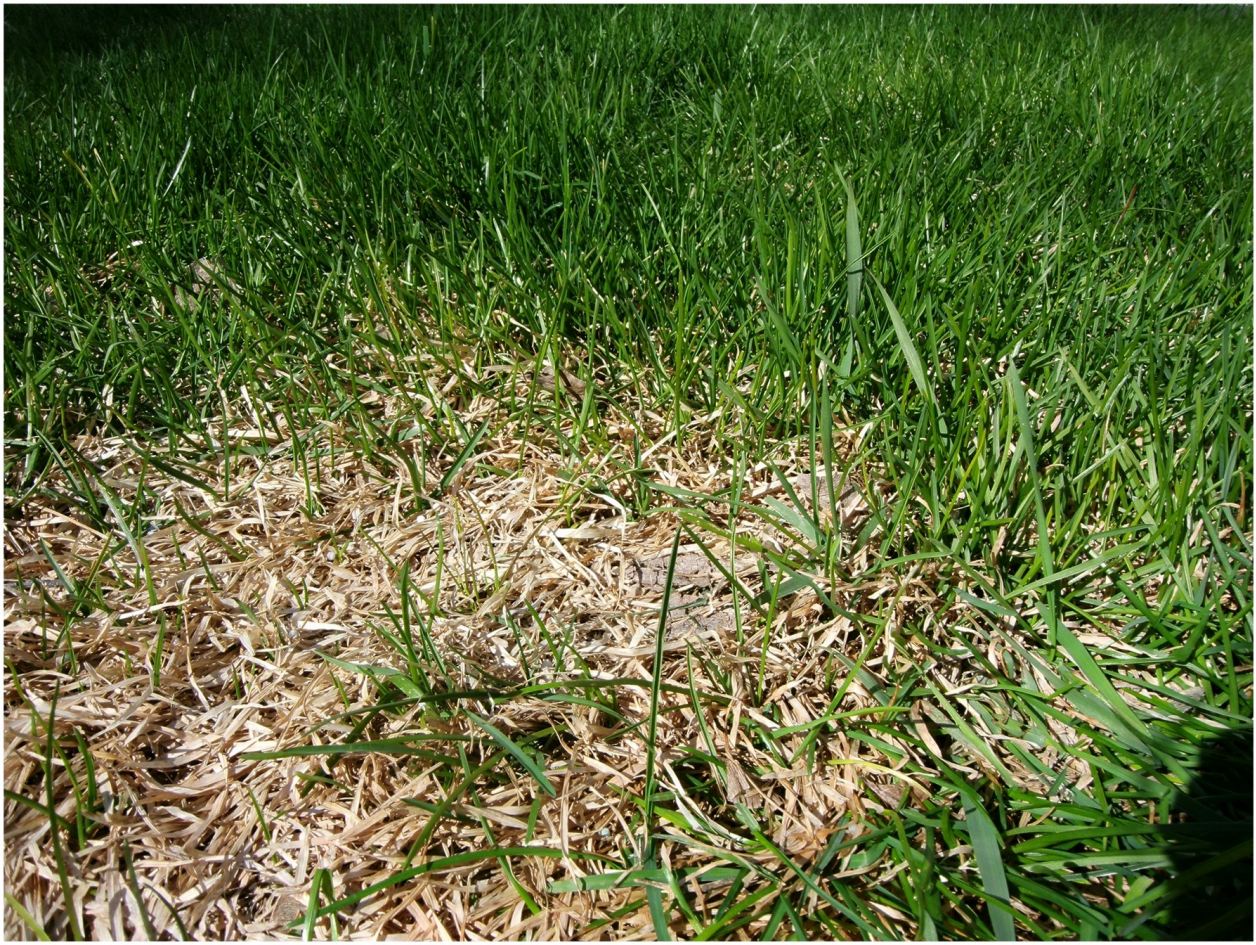
Feeling like dead grass.

The youth felt on unequal footing with those they considered “normal” as reinforced by this photo (Fig 1. Feeling like dead grass) and its associated quote by a 16 year old female.

*I think it kind of shows how with anxiety you kind of feel like especially ‘cause like I’ve said a million times it disables you to do some things*. *So you kind of feel like you’re not living the way that you could and the way that everyone around you is*. *So it’s kind of like showing that the dead grass is the person with anxiety and then the living grass all around them is like everyone else and like how they see it and you feel like jealous because you wish that life was like easier for you and how it’s like easy for everyone else or easier or whatever*…

### The struggle between being there for others and being-there-for-oneself

Youth in this study demonstrated a profound sense of responsibility, responsibility as “being there” for the other [45]. They were attuned to those around them, often to an extent wherein they could “catch” the feelings of others. Their desire to be-there-for-others was accompanied, however, by a neglect of being-there-for-oneself. As such there was a tendency for them to “feel worse” after being-there-for-others, as well as in their confusion around what it means to attend to their own needs and wants. In this way, youth seemed to be sorting out what it means to be with oneself relative to the ethical experience of the other’s vulnerability.

*When anyone I know I’m talking to is upset for any reason it makes…then I’ll just try to help them*, *but it makes me feel worse*. *When I forget to do something important or I forget to call somebody*, *I guess I feel really guilty about that*, *because it’s my responsibility and I’m not screwing it up*.

Female, 16 years

*I think being a teen in general just like very toxic cause you don’t know how to express yourself*, *you don’t know what you want and need*, *like I just know it’s really hard for my mom who tried to be there for me and I just didn’t want her and I couldn’t tell her what I needed or what I wanted because I didn’t know what was even going on*…

Female, 19 years

This is not to suggest that the experience of being-there-for-others was in every instance energy taking. Youth described times where the act of giving lead them to feel joy, or at least a temporary sense of relief from their own life’s circumstances:

*If my friends are upset*, *it puts me in a bad mood*. *And there was some days where even if I knew they had a rough night or a rough morning*, *I would stop at Starbuck’s on the way to school and buy them a latte and then just like the idea that momentarily they were happy*, *like made me feel a lot better*. *I think the most important thing for me is like for others to be happy rather than myself because like I feed off other people’s energy*.

Female, 17 years

Although youth struggled with being there for themselves, they were curious about what the act really meant for them. In some cases, it was in the neglect of being-there-for-oneself that youth made strides towards a new way-of-being. One youth, for instance, learned the importance of taking time out for herself, a method of care that prevented her from becoming “frazzled” by life’s everyday dilemmas. In her words:

*Having time gives me more time to remember things*, *like I’m always afraid I’m going to forget something*, *I’m pretty forgetful*. *It gives me more time*. *And I’m not going to be frazzled more if I go the wrong way*, *if I get lost*, *if I can’t find a parking spot*, *anything like that…I like having like a little spare time to make sure I don’t have to be rushing*, *‘cause if I’m rushing then it just makes me frazzled*.

Female, 16 years

It seemed that contact with people who shared the condition of anxiety (or a related condition) served as a refuge or sanctuary for them, a space where they could attempt to find a balance between being there for others and being there for themselves. This balance could be found in a community where youth could be away from the presumptions of others, surrounded instead by people who were compassionate, and were more or less like themselves. In some cases, encounters with others who suffered in a similar way helped youth find meaning in their experience, something that was difficult to achieve through introspection alone, and contributed to their recovery.

*When I volunteer at the [community organization dedicated to working with mental health disorders] I’m dealing with people who live with it…And they’re like my best friends in a way*. *So I just love to be there and I love to be surrounded by like everyone to me is I know using the word ‘normal’ isn’t you know a good word but I think everyone’s the same…they are equal to me*…

Female, 22 years

### Discovery of the self

The youth in this study were engaged in a process of self-discovery, a process that was continually emerging [52]. Anxiety presented a unique dilemma to the youth’s discovery of self. It seemed to leave them conflicted about whether anxiety was part of the self or an entity foreign to the self, a conflict that may have in part been influenced by stigmatizing social constructs [53].

A phenomenology of the body would suggest that, “The conspicuous disturbance [anxiety in this case] always possesses the character of an encumbrance…something that stands before us as it were…the experience of object, the disease as entity… it is when this relation remains disturbed in a disquieting manner that we exist in a protracted state of ‘dis-ease’…” [44] (p. 6). This notion becomes apparent through the youth’s accounts, in that the experience of those who struggled to “find a place” for anxiety within the self, seemed to exist in a state of psychological dis-ease as noted by the following account:

***Youth***: *When it [anxiety] happens you’re the opposite of who you are…You’re the exact opposite*, *you yell*, *you scream*, *you hate everything in your life…It’s hard to go against*.

***Interviewer***: *When it’s [anxiety] not there…how would you describe you*.

***Youth***: *Well sometimes I can be a bit shy…I like to be myself a lot…I like to laugh and I love to be happy and cheerful to everybody and I love*, *I just love to be sunshine*, *it’s my favourite thing…But*, *but then anxiety comes in and it changes me the exact opposite*. *Like* ***y****elling*, *slamming things*, *nearly breaking everything*.

Female, 10 years

**Fig 2:**
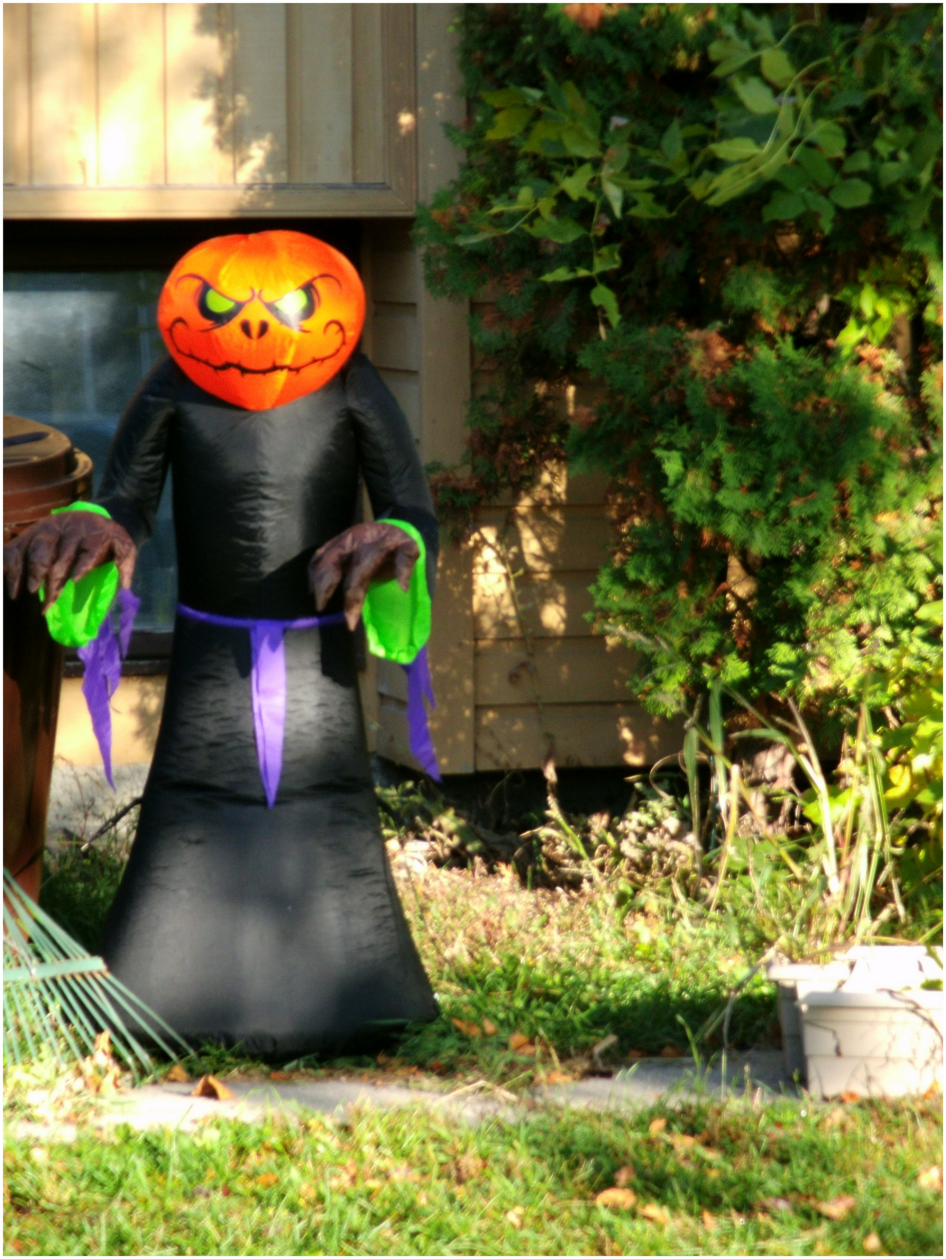
The scary monster.

Another 19 year old female youth described the sentiment in one of her photos (Fig 2. The scary monster) and associated quote:

*This one is a picture of what anxiety kind of sort of looks like in my head…It looks like a monster…I don’t know it just looks like a monster*, *so that’s good…Especially when you’re not*, *you don’t have any skills to deal with anxiety yet*, *it’s just a big bad scary monster…And it depends on the day you ask me*, *sometimes anxiety feels like a monster inside of me and sometimes anxiety*, *if I go without anxiety for a long time it will be like the monster is coming back to me*.

Outside of the immediate experience of anxiety, youth referred to certain qualities that were characteristic of the self; among the most commonly noted were a heightened sensitivity and a tendency toward introversion. Coming to learn about these parts of the self often occurred within an intersubjective space, and appeared to involve a negotiation between the opportunities and challenges of parts.

*I’m a very sensitive person…not that I’m not sympathetic it’s just like it’s hard for me…It’s weird*, *like I used to be that person where it’s like come talk to me like I’m here*, *but now I don’t know why I’ve almost like closed myself off towards like other people in a way*, *where it’s like very hard for me to like make friends…but now it’s hard for me and when people like come to me with problems*, *I just don’t know what to say…I mean it can come in handy but it can also be very hard to deal with to be very sensitive*.

Female, 19 years

### Masking the self

The manner of the look of the other, whether confirming or criticizing, objectifying or subjectifying, has the potential to rob one of their subjectivity, in some cases producing a self-consciousness or a reduction in one’s sense of self [47]. This notion becomes important in the case of illness where a “sick body” is often subject to the objectifying look of others [44]. It was not difficult to see that youth in this study sensed their vulnerability to the look of the other, and were intentional about their expression of anxiety through “masking of their true self.” Youth became rather astute at determining how the other might qualify them. The concern for masking the self was reiterated by youth who masked their true self so as not to be a burden on others in their lives:

*Well sometimes*, *I’ve never thought of it as hiding but sometimes I just think well better not tell my mother about it [anxiety] because it probably won’t help anyone and I know what she’s going to say anyways and that probably won’t help me*.

Male, 13 years

***…****like as soon as my mother sees me*, *she worries about it*. *She actually does not sleep*. *Like she will stay awake*, *she’ll actually come up to you in the middle of the night waking you up*, *say ‘are you okay now*?*’…So come to work*, *everything’s right*, *fine*. *I have the fakest*, *like I put a mask in front of her and it’s not me*. *I try to hide from my siblings as well*, *my brothers*. *They’ve also got enough to deal with*, *but for my mom I always have to have a smile…I’ve seen it enough where she saw how anxious I was of something*, *worried*, *it wasn’t a good day for her*…

Female, 22 years

**Fig 3:**
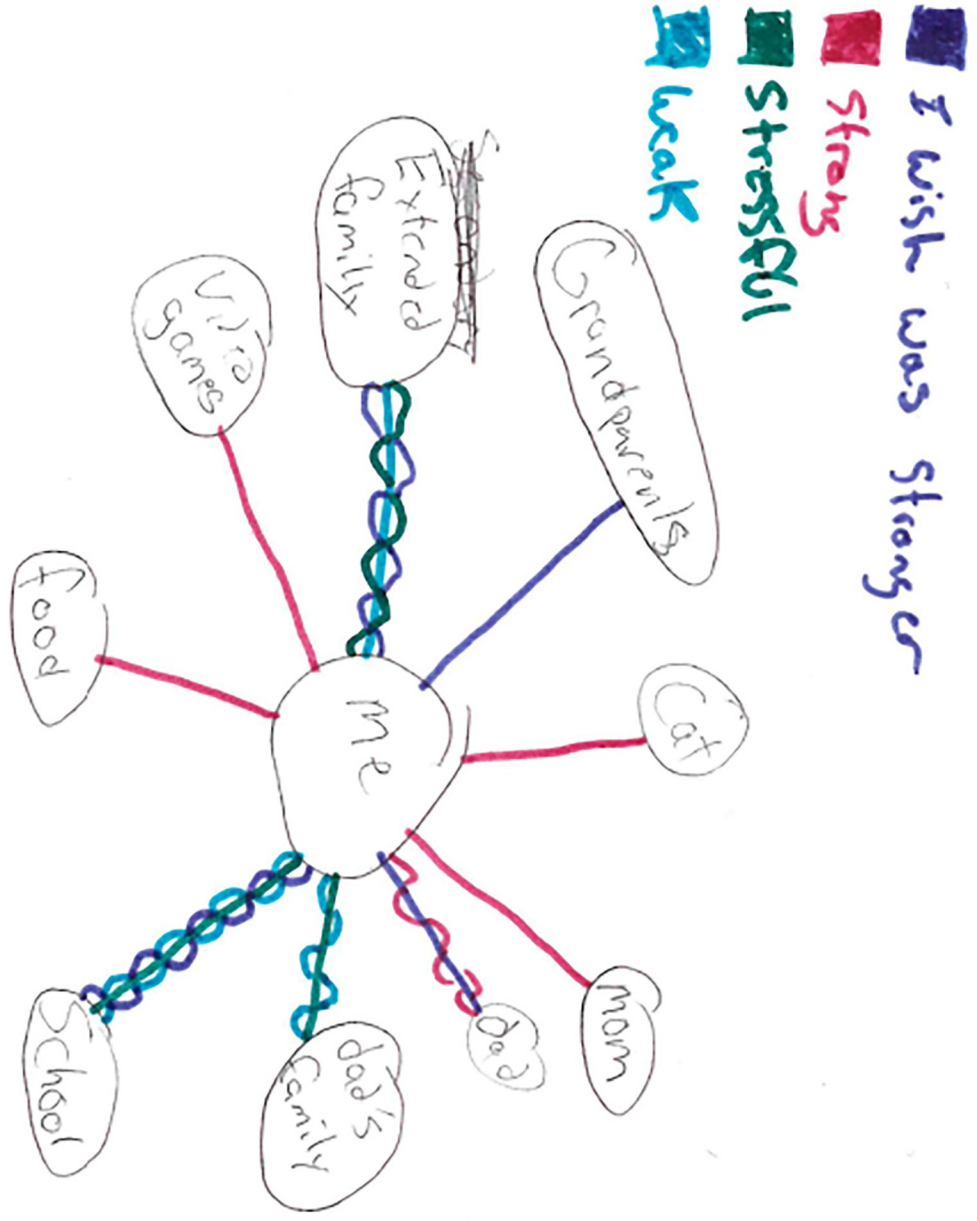
Ecomap depicting fractured relationships.

Masking of the self often resulted in fractured relationships between youth and the significant people in their lives as reinforced in an ecomap (Fig 3. Ecomap depicting fractured relationships) drawn by 16 year old male who spoke of fractured relationships with his father and his father’s family, extended family, and peers at school.

Situations were presented to the youth in which their masking was detrimental to the self. Youth described how this tendency could leave the other confused, frustrated, and unable to help. The work involved in trying to maintain the mask at times led youth to become defensive, and “get really cocky” as one 17 year old female noted. In other situations, wearing a mask in public spaces could serve to “trick” youth in that they would start to take on characteristics of the mask, however the work involved in masking was not always sustainable. Let us consider the following account:

*Um for example when I wear it [mask to disguise anxiety] for my mom I know that I want to take it off as soon as I can…But when I’m having a rough time at home and I go to school or I meet people in work I put on my mask and I have it on long enough that it does trick me…that mask almost becomes my actual face*. *Until I get home and it’s almost like I snap back into reality and I realize the whole day I was smiling acting all peppy*, *but in reality I wasn’t good*.

Female, 22 years

### Trust in the self

The youth appeared to struggle with trusting themselves, an experience that expressed itself through a relentless amount of questioning and self-doubt. Asking questions in one’s life holds tremendous value, in that it can allow one to move beyond a “self-forgetful, passed-over relation” to the self [40]. However, youth’s uneasiness towards their questioning seemed to suggest that they had not found a place for doubt and uncertainty in their experience of the self [54]. That is, doubt at this juncture in their lives was more of an encumbrance and hence a source of dis-ease; they were especially uneasy about making the wrong decision, as well as how they might be perceived through the eyes of the other.

**Fig 4:**
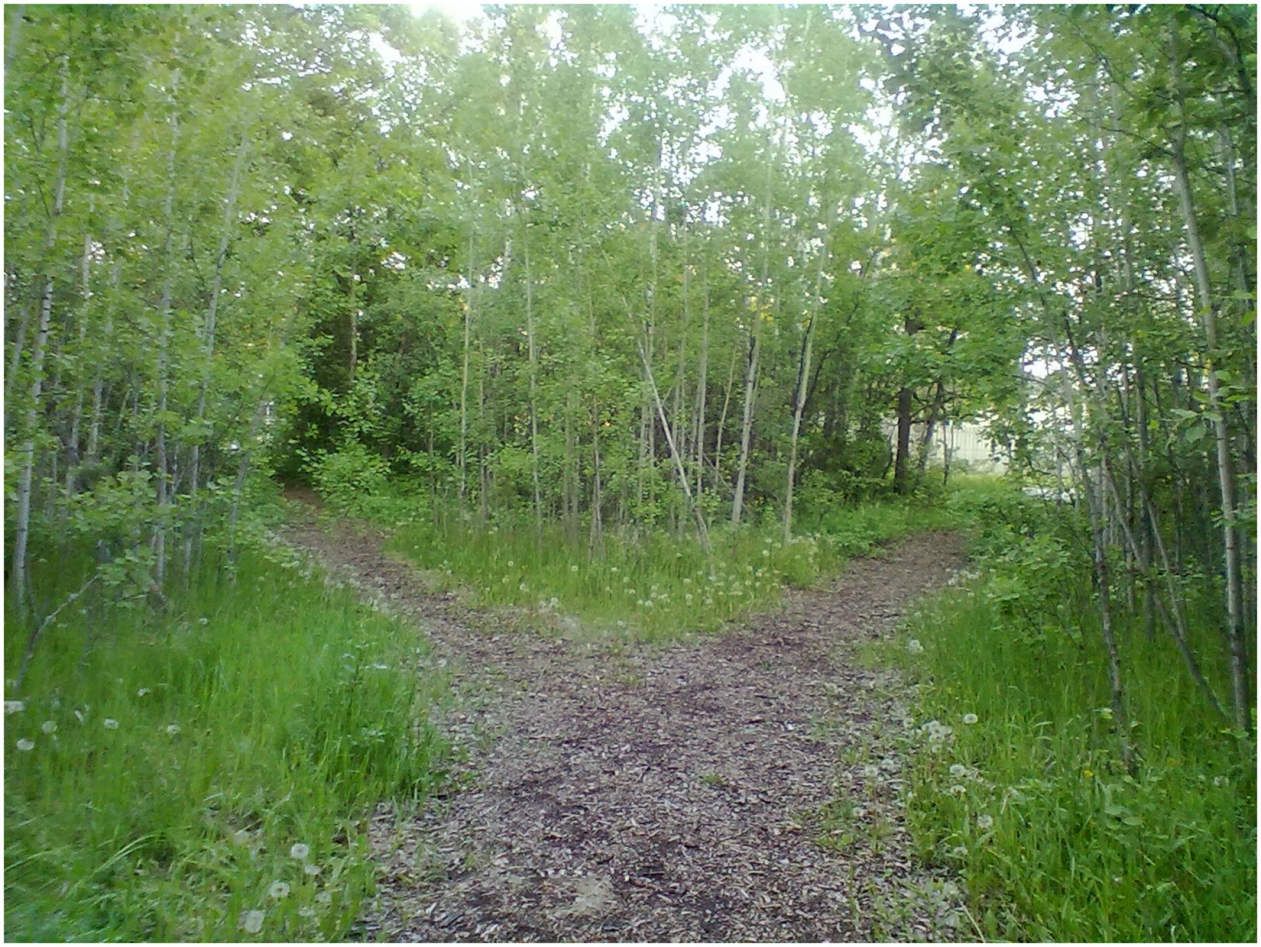
Two pathways.

*And I always take a picture of this*, *if you notice there’s two pathways (see* Fig 4. *Two Pathways)…So even though I’m going out to nature I feel better*, *as soon as I approached this almost like a fork right…I start feeling anxious again…Like what way do I take*? *Which always represents me in life*, *whenever I’m facing a decision it’s one of the worst causes of my anxiety because the what if comes up…What if I take the wrong path and get lost or end up you know causing more problems or you know so many things come up*, *even though it’s such a beautiful scenery and it’s supposed to make me feel good*, *I stood there going*, *‘which way’*? *I mean even though it’s no big deal*, *I’m just taking a walk*, *all of a sudden I just thought of wow*, *like anything that gives*, *makes me decide…*.*It just it feels horrible inside…*.*I hate deciding*.

Female 15 years

***Interviewer***: *How would you describe to someone what your daily life is like*?

***Youth***: *Well I guess it’s pretty stressful…Oftentimes when I’m in public I’m like just be worrying what other people think or whatever*!

Male, 16 years

**Fig 5:**
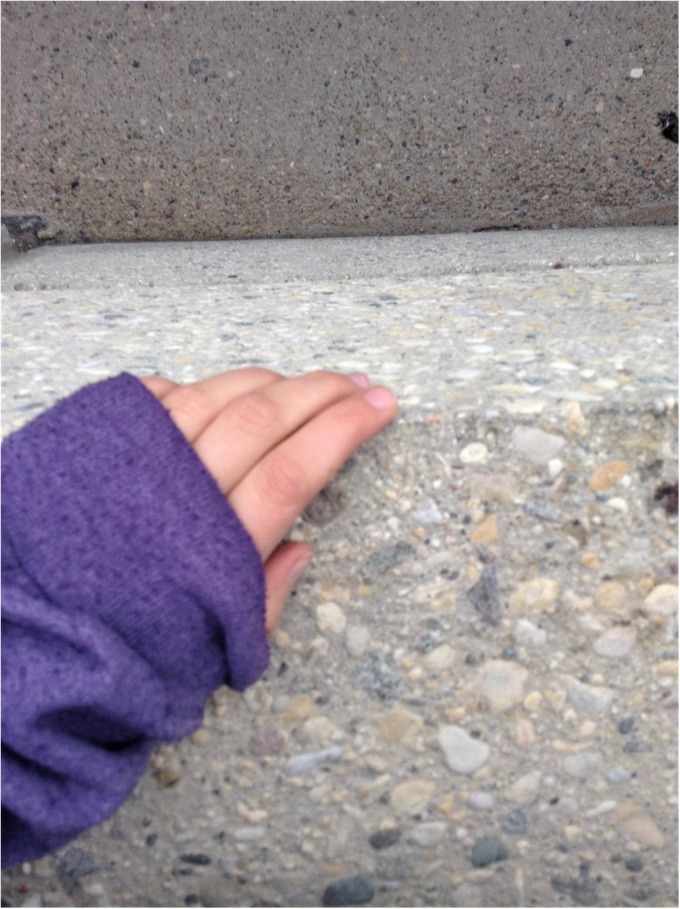
Trust yourself.

It appeared that moments of utter devastation pushed some of youth to an awareness of the importance of trusting themselves. One youth describes her transcendental encounter with an earlier desire to physically harm herself:

*I was sitting on this ledge…and I took this picture (see* Fig 5. *Trust yourself)…and I guess the idea that there’s so many opportunities to harm yourself…****…****like I can stay away from knives so I know I won’t stab myself or cut myself but sitting outside a building on a ledge… and it’s like*, *I can’t protect myself from everything and then it kind of taught me to trust myself more because it’s like care for myself more because it’s like if you really wanted to do it you have ample opportunities to…*.*just the amount of trust you have in yourself was like it kind of like struck home for me and I was like*, *like you could literally kill yourself at any time with anything anywhere…and just the idea that I always have to care for myself and trust myself to not let my myself do something like that and it’s not as easy as just staying away from knives*. *It’s doing anything*, *you have to trust yourself and care for yourself and it just kind of like took something like this to remind myself that it’s not necessarily anything explicitly dangerous that you could harm yourself with*, *it’s like you can turn anything into a weapon so*.

Female, 17 years

### The transcendental self

As youth struggled with the self in anxiety, it appeared that they were able to reflect on the self and transcend earlier ways-of-being through *awareness*. van Manen [44] suggests the “…person is challenged to develop a livable relation with his or her body in the world… we must constantly be reflectively engaged in questioning how to live in contextually appropriate relations with the body and how to acknowledge the ultimately mysterious nature of embodied being such that possible inspirited body relation may be brought into view” (p. 17). In the youth’s accounts, self-reflective attitude and now awareness of certain tendencies such as taking things too personally, and becoming overly worried about certain matters were evident. In their own words:

***…*** *like my phone causes me anxiety sometimes*. *So sometimes if I am texting or trying to get a hold of my boyfriend and he doesn’t respond I’ll get very anxious and say ‘oh what’s he doing*, *what’s wrong*, *why isn’t he you know*,*’ like you’ll have thoughts come into your head and those thoughts can lead to sadness and that’s why like I just prevent them now*. *Like he doesn’t respond I never think that he doesn’t like care about me or that he’s ignoring me*, *but I could see how it would make someone feel that way and other people think like that’s so ridiculous that you’d feel that way…like that’s how I feel and that’s how it makes me feel*, *so don’t judge me on this*.

Female, 19 years

*I don’t know why but*, *it happens*. *I’m glad and not glad at the same time*. *Like I’m glad that I’m having this like getting worried because I wasn’t before*. *Now that I know that I worry about stuff more like I’m able to catch it…I’m glad that I can and that I can improve it*. *I’m not glad because I’d like to be just like calm and relaxed about most things most of the time and I sometimes can’t be*. *But I don’t and especially I don’t know why I have it*.

Female, 16 years

The possibility for transcendence was further made possible through the perspective youth took on their experience of anxiety. They perceived in some cases that their anxiety made them stronger, or allowed them to experience the world in a way that those without anxiety could not. In all this, it is interesting to note that while anxiety allowed some youth to transcend earlier ways-of-being, they were acutely aware of its potential to lead them astray.

*I guess anxiety can look beautiful*. *And it can give you two sides of a scenario; it can bring you closer or push you away…Change you in an instant almost and you’re still the same person like*. *I guess because anxiety has taught me a lot about myself…And has taught me a lot about other people and their struggles to with anxiety that I would not have known if I didn’t confront this*. *So it changes the way you think and it changes the way you see things*. *And it opens your eyes*, *but at the same time it can close them*.

Female, 20 years

***Interviewer***: *If you could change anything about yourself would you*?

***Youth***: *Hmm*, *well you know I think maybe most people would answer that*, *maybe say that they don’t want anxiety anymore*, *they just want it to go away*, *change that about themselves*. *I don’t think I would change anything…I know its strange (chuckle)because it makes who I am today and I can’t see myself in any other way…I just*, *I’m me and this is who I am so (chuckle)*.

Female, 22 years

Anxiety appeared to stimulate a “generativity” in youth, in that they demonstrated a striking sensitivity to the suffering of the world and others [55]. They expressed multiple concerns (e.g., concern for the excessive waste produced by the food industry), they felt compassion for others (e.g., compassion for the troubled histories endured by Indigenous youth in Canada as a result of colonization), and they wanted to help others who were in a similar situation as themselves, struggling with psychological dis-ease:

*I even told my psychiatrist this and I told my counsellor and my clinical psychologist this*, *I feel it might sound funny but it’s a blessing to be diagnosed with these things*. *Because I hope to be a professional in the future and when you’re sitting in front of a patient or a client or whatever*, *and they’re telling you about their problems*, *I feel to really connect with them and to really help them is to actually have walked in a similar path*. *No one could ever actually walk in your shoes…It’s very unique what you feel*. *But to sit there when someone tells you know…I*, *I’ve been through similar things*. *I feel like it helps so much*. *And just to tell someone you know that person’s struggling*, *I’ve been through something similar*, *this is how*, *this is how the motions are; this is what I’ve learned****…****You know maybe we can help them by this way or that way because I*, *I’ve survived right…So I guess it’s really*, *it can be really good; I guess that’s what I mean by anxiety being a good thing in a way…Because I can say you know you have anxiety*, *I have anxiety*, *let’s hold hands and get through*.

Female, 22 years

## Discussion

From a hermeneutic phenomenology approach, the purpose of this research was to deepen understanding of the lifeworld of youth diagnosed with an anxiety disorder. The study revealed that youth in this study suffered a great deal in that the self, the center point of experiencing, appeared to be “fractured,” setting up for a great deal of self-scrutiny and a lack of self-compassion. In this way, youth experienced a profound sense of responsibility for the other but were neglectful where it concerned being-there-for-oneself. Navigating the social world presented an additional challenge as youth were sorting out where, and in whose presence it would serve them to mask the self. In all this, youth were genuinely interested in self-discovery and transcending earlier ways-of-being through awareness and other means.

The findings mirror earlier phenomenological investigations that similarly reveal the experience of a fracture in anxiety-affected youth, though described differently in other studies as a “fundamental flaw” or “worthlessness” [14, 23, 56]. The present study builds on this work by showing how the experience of a fracture can lead youth to wonder about their existential condition and question *what* they are, something Kazanjian and Choi [57] recognized in their humanistic reflections of the anxious child, and saw as an important aspect of “creatively overcoming” (p. 44). It is fortunate that youth in the study could take refuge from their rather relentless self-scrutiny in communities of people who suffered in a similar way, an aspect of healing that the existential-phenomenological explorations of Laing [58] pointed out many years ago.

As one might suspect in the case of a fractured sense of self, youth in the study experienced a neglectfulness where it concerned being-there-for-oneself. They were nevertheless able to “be there” for others, an experience that left them feeling depleted in some cases and nurtured in others. On the surface, this finding appears to contradict earlier work that describes anxiety-affected youth as “…too absorbed in their own world” [31, 56]. However, youth in this study were both excessively focused on themselves (though not necessarily being there for oneself) while also being preoccupied with others.

Youth’s experience of the other was complicated further by how they anticipated others to react to their anxious feelings. There is evidence to suggest that youth living with anxiety are commonly stigmatized by their family, peers and school staff [59], which could explain why youth in this study may have guard against through a “masking” of the self. In line with Hjeltnes et al. [23], the findings showed that while “putting on a mask” protected these youth in certain relational encounters, it simultaneously created dilemmas including youth spending a considerable amount of time and energy looking inwards as well as others not knowing how to help them.

It was fortunate that youth not only remained curious about what it meant to be-there-for-oneself, they also showed a genuine desire for self-discovery. Kanzajiaan and Choi [57] suggest that youth experiencing anxiety do well to remain curious about their condition, because it is in their questioning that “they will emerge as a person” (p. 46). This is not to be confused with the habitual questioning and self-doubt of these youth, which was also revealed in this study. Rather, youth in this study were curious about their own personal qualities (e.g., heightened sensitivity) and were especially curious about where to place the anxiety they felt in their experience of the self. It seemed that those who were able to accept anxiety as part of the self, experienced less psychological dis-ease than those who were not able, a finding that is supported by a holistic approach to care that places acceptance (and awareness) of the self at the heart of recovery.

A transcendental quality emerged through the youth’s voices. Anxiety has long been credited for its ability to propel one to a heightened state of self-awareness if it is approached in a certain fashion [60], a theme that was clearly reflected in the study. It was through their experience of anxiety that youth were able to develop greater trust in themselves and reflectively engage their past, cultivating an awareness of self, a new perspective on their suffering. Youth in the study were highly sensitive and empathetic to the experiences of others, rather than to say the devastation in the world. Only a few studies have explored the experience of transcendence in anxiety-affected youth [14, 29] reinforcing the need for more work in this area.

Care and support to youth with anxiety requires an understanding of the ways in which the self may have been fractured by their experiences with anxiety. Providing young people with an opportunity to share with others who had similar lived experiences can serve to contribute to a sense of healing for youth, while also providing a safe space in which young people can let down their guard and openly acknowledge or share their experiences without fear of stigmatization.

## Limitations

A number of limitations are worth noting. First, even though the goal of purposive sampling is to maximize diversity [35], the majority of youth in this study were white and female, limiting phenomenological explorations to certain social and cultural conditions. Second, the study, to the degree possible focused on the condition of anxiety in young people, and hence future investigations that considers emotional and behavioural challenges more broadly will likely produce alternative perspectives. Third, the voices represented here were entirely those of the youth themselves; drawing on the perspectives of family members, teachers and psychological care workers will provide context to future investigation. More research is also needed in order to understand the developmental differences that may exist between specific developmental groups.

## Conclusion

The phenomenological accounts by youth on living with anxiety reinforce the challenges they experienced within themselves that give rise to a great deal of inner turmoil. However, it is hopeful that they are committed to a transcendence of the self, whether through awareness or adopting a new perspective. In this spirit, those who share relationships with these youth are encouraged to listen deeply for these narratives, foster the space for youth to engage in a process of self-reflection, and offer support to youth as they embark on their journeys toward self-discovery.

## Supporting information

S1 Interview Youth open-ended interview guide. (DOCX)

S2 Interview Youth photovoice interview. (DOCX)
