# Supplementary material for: The experience of the self in Canadian youth living with anxiety: A qualitative study
Source: PLoS One. 2020 Jan 31;15(1):e0228193. doi: 10.1371/journal.pone.0228193 (PMC6993971; doi:10.1371/journal.pone.0228193)
Supplement: S1 Interview — (DOCX) [file pone.0228193.s001.docx]

Youth Open-ended Interview Guide: S1.Interview

**Introduction (Background Questions)**

1. Could you please tell me a little bit about yourself?

The interviewer will ask additional questions that touch on familiar territory for the youth (e.g., general questions about school, family, favorite activities). The ecomap will be introduced here to facilitate the youth’s responses. The interviewer will then make use of the youth’s ecomap throughout the interview when appropriate.

- Could you please tell me a little bit about your family; parents; and brothers/sisters?
- Could you please tell me a little bit about your friends?
- Could you please tell me about your school?
- Could you please tell me about any other people in your life who are important to you?

Once the interviewer feels that the youth is comfortable, the interviewer will then proceed with question #2.

**How Living with an Anxiety Disorder Shapes Daily Life**

2. A lot of young people have problems with nervousness, worry or fears. I was wondering what areas have been difficult for you. Could you please tell me more about that?

- Could you please tell me what it is like to have a problem with ----------- (e.g., fears or worries)?
- Could you please tell me about the types of symptoms (or word that the youth is familiar with) that you experience?
- What feelings do you experience or have in your body?
- What worries do you have when you are feeling really nervous or scared?

**How Living with an Anxiety Disorder Shapes Daily Life**

3. In general, could you please tell me how having an anxiety disorder or your problem

with ---------- (term youth uses) affects your life?

- How much does it influence/affect your daily life?
- How do you spend your day?
- What are some of the challenges (good and bad) that you face?
  - Could you please you give me an example of a good day?
- Could you please you give me an example of a bad day?

4. Could you please tell me how having an anxiety disorder or your problem with ---------- (term youth uses) affects how things go between you and people in your family?

- Could you please tell me how having an anxiety disorder or your problem

with ---------- (term youth uses) affects the types of activities that you take part in with your family; parents; and brothers/ sisters?

5. Could you please tell me how having an anxiety disorder or your problem with ---------- (term youth uses) affects how things go between you and your friends?

- Could you please tell me how having an anxiety disorder or your problem

with ---------- (term youth uses) affects the types of activities that you take part in with your friends?

6. Could you please tell me how having an anxiety disorder or your problem with ---------- (term youth uses) affects how things go between you and any other people in your life who are important to you?

- Could you please tell me how having an anxiety disorder or your problem

with ---------- (term youth uses) affects the types of activities that you take part in with them?

7. Could you please tell me how having an anxiety disorder or your problem with ---------- (term youth uses) affects how things go in school?

- What do like best about school? What do you like least about it?
- Could you please tell me how having an anxiety disorder or your problem

with ---------- (term youth uses) affects how things go between you and your teachers at school?

- Could you please tell me how having an anxiety disorder or your problem with ---------- (term youth uses) affects the types of activities that you take part in at school?
- What challenges have you had in school? What opportunities do you have in school?

8. Could you please tell me about some of the other activities/situations/events (other than activities/situations/events that you previously talked about) that you like to take part in?

- What activities/situations/events would you like to take part but do not?
  - What activities/situations/events do you avoid or try to avoid?
- What activities/situations/events do you do that you think are not good for you?
- What activities/situations/events do you do that you think are good for you?

9. Are there any other experiences that you would like to share with me that you think will help me understand what it is like to have an anxiety disorder or your problem with ---------- (term youth uses)?

- Describe some of the good things that have happened to you.
- Describe some of the bad things that have happened to you.

10. Could you please answer the following question: In general, I am -------------about my everyday life? Please tell me why you answered the way you did.

- What things in your life make you happy?
- What things in your life make you unhappy?

**How Youth see themselves within the Context of Living with an Anxiety Disorder**

11. If your (close friend, parent etc.) asked you to describe yourself, what would you tell them?

- If your (close friend, parent etc.) asked you to tell them what you are like, what would you tell them?
- How do you think your (close friend, parent etc.) sees you?
- What do you like best about yourself? What do your friends, close friends etc. like best about you?
- What do you like least about yourself? What do your friends, close friends etc. like least about you?

12. Could you please answer the following question: In general, I am -------------about myself?

Please tell me why you answered the way you did.

- Five years from now I see myself----------? Five years from now I would like to be----------?
- If I could change anything about myself it would be----------?

**What Hinders and what Helps Youth with an Anxiety Disorder to Live Satisfying, Hopeful, and Productive Lives**

13. Could you please tell me about the things you do that help you cope with your anxiety disorder or your problem with ----------- (e.g.., fears or worries)?

- What are the most helpful things that you have done that help you to cope your anxiety disorder or your problem with --------- (e.g.., fears or worries)?
- What things make living with an anxiety disorder (or your problem with…) seem not as bad?

14. Could you please tell me how others (e.g., **probe on** family, friends, teachers, and other people important to the youth) have tried to help you cope with your anxiety disorder or your problem with ----------- (e.g., fears or worries)?

- What are the most helpful things that others have done?
- What are the least helpful things that others have done?

15. a. What things do you do that help you to live a satisfying life?

b. What things do others do that help you to live a satisfying life?

16. a. What things do you do that help you feel hopeful?

b. What things do others do that help you feel hopeful?

17. a. What things do you do that help you feel a sense of accomplishment?

b. What things do others do that help you feel a sense of accomplishment?

18. If your life was the best it could possibly be, what would that be like?

- What would help to make your life better?

19. What advice would you give to other people about how they could help youth cope with their anxiety disorder (or term youth uses)? Please have the youth share with you what he/she would say to parents, siblings, friends, and other people important to the youth.

- What advice would you give to others that would help to make your life better?

20. What advice would you give to other youth to help them cope with an anxiety disorder (or term youth uses)?

- What advice would you give to them that would help to make life better for them?

**Ending Questions**

21. Can you please tell me what made you decide to take part in this study?

22**.** Is there anything else you would like to talk about that we did not talk about?

Are there any other questions we could ask youth participating in this study that you think would be helpful to this study?
