# Supplementary material for: The experience of the self in Canadian youth living with anxiety: A qualitative study
Source: PLoS One. 2020 Jan 31;15(1):e0228193. doi: 10.1371/journal.pone.0228193 (PMC6993971; doi:10.1371/journal.pone.0228193)
Supplement: S2 Interview — (DOCX) [file pone.0228193.s002.docx]

Youth Photovoice Interview: S2.Interview

**Preamble:** Prior to asking questions about the photographs, begin by asking the participants how they have been since the last interview. This should then be followed by asking them if they have anything to add to about what they said in the first interview. After this they can then talk about the photos.

**“SHOWED” Framework** (Adapted from Dahan et al., 2007)

| **S** | What is **S**een here?   - Describe what the picture is of. - What is this picture about? |
| --- | --- |
| **H** | What is really **H**appening?   - What is the story behind this picture? |
| **O** | How does this relate to **O**ur life (or My life personally)?   - Describe why you decided to take this picture. - Could you please finish this: Taking this picture made me feel…? - What does this picture mean to you? - How did you feel when you took this picture? |
| **W** | **W**hy are things this way?   - What are the reasons that you think this exists? |
| **E** | How could this image **E**ducate people about what it is like to be a youth with an anxiety disorder?   - What is the main message behind this picture? - What would you like to share with others (e.g., family, parents, sisters and brothers, friends, doctors, therapists etc.) about this picture? - Is there anything else that you would like to tell others about this picture? |
| **D** | What can I **D**o about it? (What will I or WE do about it?)   - What should be done about this? - What could be done about this? - Is there anything that you would like to change in this picture? Please explain. |

Dahan R, Dick R, Moll S, Salwach E, Sherman D, Vengris J, Selman K. Photovoice Hamilton: Manual and resource kit. Hamilton, ON.: Photovoice Hamilton. 2007.
